# Towards the Segmentation and Classification of White Blood Cell Cancer Using Hybrid Mask-Recurrent Neural Network and Transfer Learning

**DOI:** 10.1155/2021/4954854

**Published:** 2021-12-02

**Authors:** Sumit Kumar Das, Kazi Soumik Islam, Tanzila Ahsan Neha, Mohammad Monirujjaman Khan, Sami Bourouis

**Affiliations:** ^1^Department of Electrical and Computer Engineering, North South University, Dhaka-1229, Bangladesh; ^2^Department of Information Technology, College of Computers and Information Technology, Taif University, P.O. Box 11099, Taif 21944, Saudi Arabia

## Abstract

Inside the bone marrow, plasma cells are created, and they are a type of white blood cells. They are made from B lymphocytes. Antigens are produced by plasma cells to combat bacteria and viruses and prevent inflammation and illness. Multiple myeloma is a plasma cell cancer that starts in the bone marrow and causes the formation of abnormal plasma cells. Multiple myeloma is firmly identified by examining bone marrow samples under a microscope for myeloma cells. To diagnose myeloma cells, pathologists have to be very selective. Furthermore, because the ultimate decision is based on human sight and opinion, there is a possibility of error in the result. The nobility of this research is that it provides a computer-assisted technique for recognizing and detecting myeloma cells in bone marrow smears. For recognizing purposes, we have used Mask-Recurrent Convolutional Neural Network, and for detection purposes, Efficient Net B3 has been used. There are already many studies on white blood cell cancer, but very few with both segmentation and classification. We have designed two models. One is for recognizing myeloma cells, and the other is for differentiating them from nonmyeloma cells. Also, a new data set has been made from the multiple myeloma data sets, which has been used in our classification model. This research focuses on hybrid segmentation models and increases the accuracy level of the classification model. Both of our models are trained pretty well, where the Mask-RCNN model gives a mean average precision (mAP) of 93% and the Efficient Net B3 model gives 94.68% accuracy. The result of this research indicates that the Mask-RCNN model can recognize multiple myeloma and Efficient Net B3 can distinguish between myeloma and nonmyeloma cells and beats most of the state of the art in myeloma recognition and detection.

## 1. Introduction

Leukemia [1] is a blood malignancy that affects the white blood cells (WBCs). It is a bone marrow disease that occurs when an aberrant WBC continues to reproduce itself indefinitely. These cells do not do what they are supposed to do, which is to combat infections. As they build up in the marrow, they prevent the formation of other normal blood cells, resulting in bleeding, anemia, and recurring inflammation. The leukemic cells continue to develop because they travel through the circulation over time. They create tumors and cause damage to the organs, including the kidney and liver. The French-American-British classification classifies acute leukemia into acute myelogenous leukemia and acute lymphoblastic leukemia. Acute leukemia is cancer that predominantly targets undeveloped cells and progresses quickly. Acute lymphoblastic leukemia (ALL) is most present in children, whereas adult acute myeloid leukemia (AML) is much more common in adults, but it may also affect children and adolescents. In the United States in 2009, 32,000 individuals are anticipated to be infected with leukemia, with 45,000 dying from the disease. The ability to identify the kind of leukemia early and accurately assists in the provision of suitable therapy for that type. A complete blood count (CBC) [2] is used to detect it. If the patient's count is abnormal, a bone marrow biopsy is recommended. Slide examinations are conducted to determine if there are any leukemic cells, structural bone marrow, and leucocytes. A pathologist will notice changes in the nuclei or cytoplasm of certain cells just below a light microscope and identify the abnormal cells in their unique types and subtypes of multiple myeloma. This categorization can be used to anticipate the disease's clinical behavior, and the patient should be treated accordingly. Due to an unknown cause, the bone marrow creates a significant number of aberrant white blood cells in the leukemia illness. Pathology is used to manually identify leukemia, which takes time and money due to the growing cost of pathology instruments. As a result, for quick and precise results, an automated approach is used in this study. This approach covers (i) processing an image of a blood sample, (ii) segmenting the myeloma, and (iii) ultimately classifying the cells as myeloma or nonmyeloma.

In some previous works, a thresholding technique was used to assess the frequency of blood cells for cancer detection. The problem with this technique is that calculating the correct threshold value is time-consuming and difficult, but we have used CNN models because CNN can extract features from the images automatically. The main novelty of this work is that it recognizes multiple myeloma cells using the Mask-RCNN method, so that doctors can identify myeloma cancer cells very quickly without any advanced technology. There are only a few works in our data set where we chose to recognize multiple myeloma cells at the same time. We have also made a data set for detecting myeloma and nonmyeloma cells. The data set was made by cropping the multiple myeloma and nonmyeloma from the 85 images in our data set. For classification, we used the Efficient Net B3 model and got 94.68% accuracy. For the segmentation model, we have got a 94% mean average precision. The main motivation of our paper is that, by using our system, we can recognize multiple myeloma cells, which are responsible cells for white blood cell cancer, only from a bone marrow microscope image. This will help Bangladesh with the diagnosis of cancer. Doctors use many advanced foreign technologies that cost hospitals and patients a lot. Also, these technologies are not available in all hospitals in Bangladesh, but by using our system, doctors or patients can easily diagnose myeloma cells in minutes, which is more cost-efficient than foreign technologies. This is also our motivation for this work, as most of our country's people are poor and cannot afford modern technology. If doctors use our system to treat patients, the cost will be minimal.

We have used CNN [3,4] models because CNN can extract features from the images automatically. On computers, vision approaches need to extract necessary features manually from the image in order to use them to train their models. Extracting a huge number of features is very time-consuming, where CNN can automatically extract thousands of features very easily. The main objective of this paper is to develop a system that can do both segmentation (recognition) and classification (detection) on multiple myeloma cancers, which will help doctors identify the exact location of the myeloma cancer cell very quickly.

## 2. Literature Review

In this section, we have reviewed many papers and shown different types of recent methodologies used for white blood cell cancer detection. Multiple image processing methods have been developed for detecting multiple myeloma in medical images of patient blood by Abdul Nasir et al. [5,6]. For example, detecting cancer cells uses a thresholding technique to assess the frequency of blood cells. In this study, image processing techniques were used to count the number of blood cells in a medical image. The ratio of blood cells for leukemia is calculated using this counted value. The original image has been converted to grayscale, and an intensity threshold has been established to differentiate WBCs from RBCs. The operation is repeated with a fresh threshold value if the results are unacceptable. The WBC and RBC ratios for normal and diseased pictures exhibit a broad range of ratios, according to the results obtained using the watershed transform. Normal images have a ratio of 0 to 0.2, but unusual images have a ratio of 0 to 14 for AML and 0.3 to 2.6 for ALL. The problem with this technique is that calculating the correct threshold value is time-consuming and difficult.

In this work, Ms. Minal et al. [7] presented WBC classification and segmentation to diagnose acute leukemia. They offer the Otsu threshold technique for automatic picture enhancement and white blood cell segmentation. To differentiate myeloma from normal B lymphocytes, the K-nearest neighbors (KNN) classifier was utilized. The approach is being utilized in leukemia research to assess a public data set of 108 pictures given by Fabio Scotti to test and properly evaluate all illness cell classification and segmentation methods. A classification approach is the KNN technique for classifying nonparametric data. This technique is straightforward yet efficient, and it may be used to distinguish blast cells from regular WBCs. The accuracy of this approach is 93%.

Adnan Khashman et al. [8] proposed the segmentation of blood cells in leukemia patients in this work. The recommended segmentation approach was tested using pictures of leukemic living cells obtained from multiple cell samples provided online by the University of Virginia's Health Center. This study's data set includes 120 grayscale images with a resolution of 200 × 200 pixels, including 30 shots of each of the four types of leukemia. This research shows that surface morphologies may be used to get two better images of the cytoplasm and nucleus regions after segmenting a leukemic-contaminated picture. The pictures of nuclei and cytoplasm were created using the stratified threshold method. Two threshold values of intensities were given, the contaminated cell's boundary was identified, and unwanted objects in the neighborhood were filtered. Overall, the categorization rate was found to be adequate in 99.62 percent of the cases.

Even though some research in cell identification has been done and segmentation from microscopic images is relatively easy, its applicability to MM-specific tasks is relatively restricted. The first research on MM detection in which the pictures were initially treated for image enhancement was by Saeedizadeh et al. [9]. Thresholding was used to identify the nucleus and cytoplasm. Following that, bottleneck and watershed algorithms were used to separate the linked cells. Finally, nucleus eccentricity and nucleus-cell ratio were retrieved from the cytoplasm and nucleus, and classification was done with a precision of 96.52 percent using the SVM classifier. Fifty digital images from bone marrow aspiration smears were processed using this approach. These images depict 678 cells, including 133 plasma cells, 256 myeloma cells, and 290 distinct bone marrow cells.

In this publication, Ghane et al. [10] provide an easy and successful approach to identifying efficient CML cells. To take photos of their data set, a Nikon1 V1 camera was mounted on an Eclipse 50i Nikon microscope with a zoom of 1000. They gathered a total of 580 pictures for their data set. All the images are saved as JPG files with a resolution of 2592 × 3872 pixels and are in RGB format. The images were generated by pathologists at the Al-Zahra and Omid hospitals in Isfahan, Iran. In the recommended technique, white blood cells were first differentiated from those collected and created microscopic images of human blood. Then, from the neutrophil series, a new mix of typical and suggested characteristics was derived. The collected characteristics were then used to classify cells into the eight categories using the newly constructed tree classifier. Finally, a five-step process was used to get the required outcomes. The suggested method's classification results were compared against the opinions of two experts at each stage, utilizing several performance measures such as sensitivity, specificity, and accuracy. According to the computed performance measures, the approach's results are quite comparable to the outcomes of an expert's diagnosis. Cohen's kappa coefficient was also obtained as a statistical metric, showing the validity of our classification approach and proving its outstanding consistency with the diagnosis results of two pathologists. The average accuracy, specificity, and sensitivity for all chronic myelogenous leukemia (CML) groups were 99.0 percent, 99.4%, and 98.3 percent, respectively.

Chan et al. [11] discussed medical image analysis using deep learning, and it is said that deep learning is a machine learning technique. The possibility of using deep learning in medical image analysis in machine diagnosis to provide doctors with decision assistance while also increasing the effectiveness and precision of different screening and therapeutic procedures has inspired fresh research and innovation initiatives in computer-aided design (CAD). Because of the small training set, they discovered that models were not rigorously verified using huge real-world test data. Secondly, they collected medical imaging data with trustworthy annotation or reference that is typical of the patient population. The collection of healthy and pathological cases using specific imaging techniques such as position emission tomography (PET) or magnetic resonance (MR) is even more challenging since only a small fraction of patients would receive these exams and access may be dependent on disease-specific procedures in different health systems. Deep learning is expected to alter CAD and image analysis in the field of medicine. The success and growth of deep learning have inspired new initiatives to develop CAD or artificial intelligence (AI) technologies for a variety of health-related technologies. Large data sets must be acquired in order to provide appropriate preparation and approval tests in order to promote robust, profound learning frameworks and autonomous evaluation using both internal and external data to assess generalization.

Deep learning frameworks for pattern recognition and picture interpretation [12] is a study that compares the sorts of learning required in the “recognition-by-parts” framework to findings from other models. Pattern and object recognition are regularly troublesome issues since parts of various examples or articles can be very comparable: having comparable properties. The epidermolysis bullosa simplex (EBS) solution to this problem entails the creation of a midway illustration stage in which an attempt to capture the transcendent properties of prepared tests by arranging them into various locations of the pattern's characteristic areas. Parts are compared to indicated nodes, and edges are compared to linkages between parts are credited and marked charts. Minds ascribe and names should be made when coordinating information with learned or known models, and the EBS framework described above does not verify for the last completely since the neural organization is quality recorded and no portion. For the following reasons, Caelli et al. [12] learned that a basic concurrent neural network model is insufficient and inconsistent with the RBP paradigm. Second, although this difficulty could be overcome, straight neural networks would still be unable to identify groups of patterns in complicated situations concurrently without first isolating suitable picture areas. Using the EBS system, we were able to circumvent these restrictions.

Image enhancement in skincare image recognition using deep learning is the subject of Aggarwal et al.'s [13] study, which suggests diagnosing dermatological disorders might be aided by artificial intelligence and machine learning. In a variety of fields, artificial intelligence and machine learning (ML) have made considerable advances. According to the findings of this article, after assembling the pictures, each image was examined and trimmed to remove probable noise. Some photos were also rejected during the image selection procedure owing to inadequate image size or improper quality. Following the aforementioned picture processing and stratification, a total of 331, 93, 131, 28, 1, and 95 photos were chosen for model building, testing, and assessment using a neural network for pimples, eczema dermatitis, vitiligo, lupus, and redness, respectively. For the testing phase, 30 pictures of each dermato symptom image were selected. After reading this study, we came to the conclusion that image enhancement resulted in the highest gain in accuracy since atopic dermatitis has the fewest supplied training pictures. As a result, image enhancement is particularly beneficial when there are just a few training pictures available. The modeling technique is currently unable to assess the severity of illnesses, pathologies, or disease progression. In the future, changes will be made to the model to predict based on skin manifestations, the seriousness, and progression of the disease.

He et al. [14] worked on image recognition using deep residual learning. We discover extensive, objective support that these residual networks are simple to tune and can gain high accuracy with high precision, as well as a DRL framework for learning the degradation issue. Here, we learned about residual representation and shortcut connections. We also learned about deep residual learning, which may be summarized as follows: Consider H as an underneath projection that will be fitted by a few convolutional layers, with *x* indicating one of the first layers' inputs. Even though both versions must be able to approximate the required functions, the ease with which they are learned may differ. If identity mappings are optimum, solvers can simply push the weights of the multiple nonlinear layers near zero to approach identity mappings using the residual learning reformulation. We also gained a better understanding of plain and residual networks. When they used Faster R-CNN as the detection technique and made changes such as substituting VGG-16 with ResNet-101, they arrived at the conclusion that the improvements can only be ascribed to better networks because both models employ the same detection implementation. We obtained a 7.0 percent gain in COCO's standard measure on the tough COCO data set, which is a 28 percent relative improvement.

Liu et al. [15] worked on machine learning technology for image recognition in which they found transportation industry picture acknowledgment innovation is applied to tag acknowledgment to extricate tags from the complex foundation, section tag characters, and build an AI nontag programmed age calculation, which might work on the proficiency of nontag acknowledgment. The variety and high age-speed of tag-preparing test sets can accomplish the motivation behind successfully preparing solid classifiers. Machine learning is a broad phrase that refers to a group of algorithms that seek to extract implicit rules from huge amounts of past data and apply them for predictions or categorization. Image recognition technology in artificial intelligence works on the idea of using a computer to analyze images and then extract information from them. The target vehicle image identification technique was chosen as the goal, and the test software infrastructure was VC++ 6.0, Matlab7, and Windows XP. In conclusion, a lot of target information was gathered, yet in the field of target acknowledgment, it is extremely challenging to get an enormous scope of viable information. This is likewise the essential issue that prevents the use of profound learning in the field of picture acknowledgment. It is important to track down a more powerful approach to doing manual information extension dependent on the first data set so that profound learning can be successfully applied.

Martínez-Martínez et al. [16] described a new technique for detecting multiple myeloma involvement of the bone marrow in sick femurs using low-dose CT imaging. To begin, an algorithm was developed to autonomously separate the bone marrow and cortical bone from 3D computed tomography (CT) scans. First of all, they did segmentation, and after segmentation, the two discrete masks and the 3D images were cropped and divided to produce two distinct femurs for each individual, a process known as spatial synchronization. Infiltration detection was then implemented. After that, infiltration and pebbling are detected. Both of the detections are done in linear time to allow real-time diagnosis. They evaluated both infiltration and pebbling detection methods in the lab and found that they had a sensitivity of 74.9 percent and 69.2 percent and a specificity of 75 percent and 61 percent, respectively.

Xu L. et al. [17] worked on medical image recognition using deep learning. Here, we look at how DNNS has evolved as a strong machine learning technique for image categorization that differs greatly from previous techniques. They concentrated on categorizing pictures as well as doing research to accurately determine the classes and positions of items inside the images. Here, we learn about the few methods of image classification, which are VGG [18], inception [19], and ResNet [20]. And also, we learn a few methods of segmentation and object detection, which are fuzzy logic [21], Faster R-CNN [22], SSD [23], and YOLO [24]. They came to the conclusion that they utilized a statistical deep learning framework for image classification, focusing on object categorization inside the image.

In Section 2, related papers on white blood cell cancer have been discussed briefly. In Section 3, we have discussed our proposed methodologies and shown the procedures for these methods. In Section 4, we have shown both of our models' performances and will discuss them briefly. Finally, in Section 5, we concluded this paper with a small summary of our work.

## 3. Method and Materials

**Figure 1:**
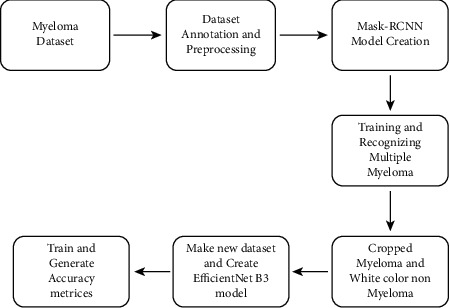
Proposed methodology of our work.

In this section, we have shown the proposed methodology of our work, the data set, and the methodologies that have been used in our work.  Figure 1 shows the proposed methodology of our work. In our proposed work, we first collect the data set from our data source and then complete the necessary data preprocessing and annotation. After the preprocessing, we need to create our first model Mask-RCNN for myeloma cell segmentation to recognize the multiple myeloma cells. For the second model, a classification model, we have cropped the purple myeloma cells and white nonmyeloma cells from the images and made a data set from them. Then, we made our classification model using the pretrained model Efficient Net B3 and trained it with our own fully connected (FC) layer. Finally, we have evaluated our two models with various evaluation metrics.

### 3.1. Data Set and Annotation

**Figure 2:**
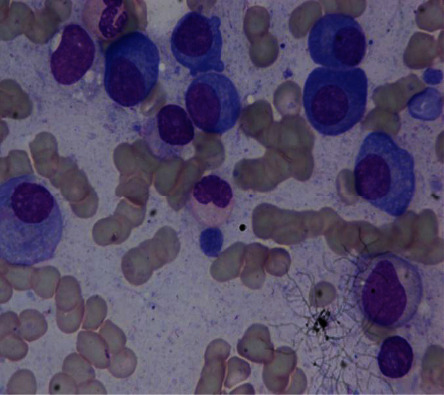
Multiple myeloma sample image from the data set.

**Figure 3:**
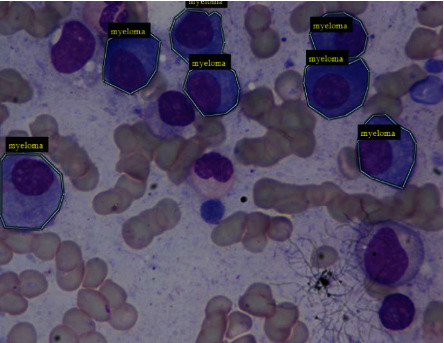
Myeloma cells annotation.

We have collected our data set from the HARVARD Blood Cancer Dataverse [25]. Using an Eclipse-200 Nikon microscope and a DSLR, images were taken at 1000x zoom. The raw BMP format with a quality of 2560 × 1920 pixels was used to capture the pictures. This collection contains a total of 85 images. All 85 images have been stained corrected and may be used to segment cells using their unique method. These stain normalized images were provided as a labeled data set on a PowerPoint slide, with plasma cells marked on all image presentations.  Figure 2 shows a sample image with multiple myeloma cells, and Figure 3 shows how we have annotated the myeloma cells for training our model.

**Figure 4:**
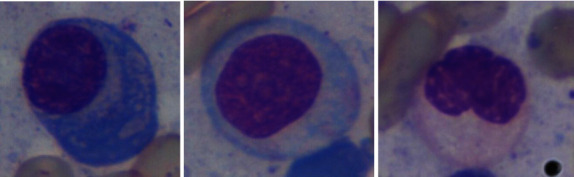
Cropped myeloma cells.

**Figure 5:**
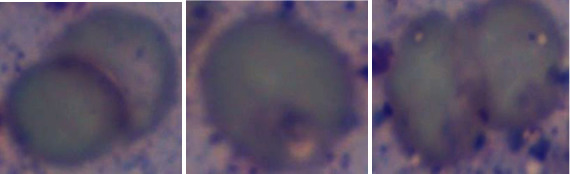
Cropped nonmyeloma cells.

To recognize myeloma cells from the image, we have annotated all 85 images. After that, for detection purposes, we have cropped all the myeloma cells along with white-colored nonmyeloma cells and made a new data set for classification where we kept 1300 images for myeloma and 1300 images for nonmyeloma cells. Figures 4 and 5 show some cropped images of myeloma and nonmyeloma cells, respectively.

### 3.2. Cancer Cell (Myeloma) Recognition Using Mask-Recurrent Convolutional Neural Network

**Figure 6:**
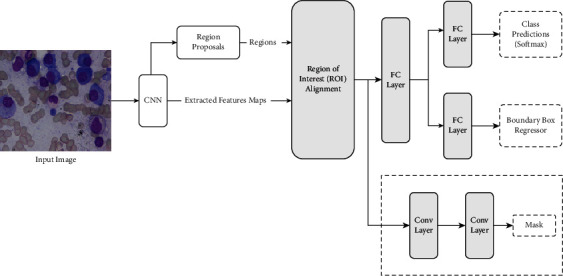
The architecture of the Mask R-CNN framework.

Segmentation can be divided into two categories. Semantic and instance are two types of segmentation. Mask-RCNN, an instance segmentation approach, is utilized in this study. In deep learning or computer vision, the Mask-RCNN is a hybrid RCNN that addresses instance segmentation problems. To put it differently, this can tell the difference between different objects in a photo or video. Giving it an image as input will give you the classes, bounding object boxes, and masks. The Mask-RCNN is split into two parts. Mask-RCNN models are trained using the COCO data set. Mask-RCNN is a cross between a Faster R-CNN (bounding box + class) for object identification and a fully convolutional network for pixelwise mask detection. The two sections are plainly seen in Figure 6. In this case, CNN stands for extracting features from the backbone. The anticipated bounding box, class name, and mask of the anticipated class are all outcomes of Mask-RCNN.

### 3.3. Cancer Cell (Myeloma) Detection Using Transfer Learning Model Efficient Net B3

**Figure 7:**
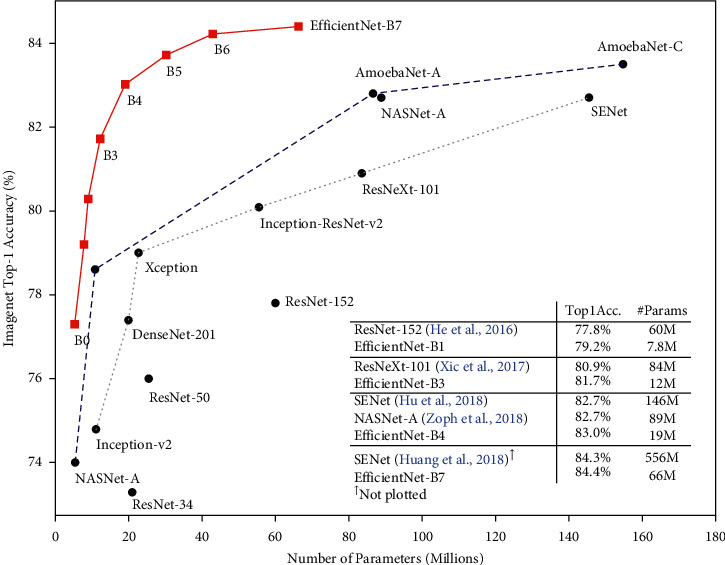
Some popular pretrained models [26].

Multiple hidden layers and max or average pooling layers are typically used in CNNs. Transfer learning was applied to identify paddy infections in this study. There are a lot of pretrained models out there that are good, well known, and have billions of images. The overall efficiency of the pretrained model is observed in Figure 7. To create a model from a pretrained model, remove the FC (fully connected) layer of the pretrained model and combine it with a new FC layer. The accuracy of all efficient net models is excellent. Despite the fact that B7 does have higher performance than B3, B3 was chosen since the necessary accuracy had already been achieved. B7 can be utilized to view the difference in future work. VGGNet, AlexNet [25], and ResNet are examples of some good pretrained models other than efficient nets.

**Figure 8:**
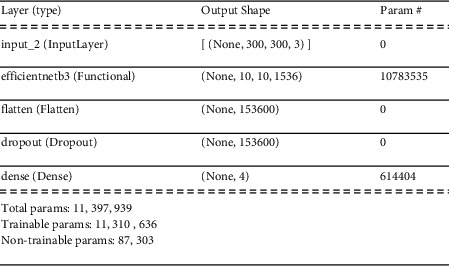
Layers and parameters of our model.

The first CNN layer is the input layer shown in Figure 8. There is also the image input size to consider, and since the pictures are 300 × 300 pixels, the input size is 300 and the dimension of the image is 3. The architecture of Efficient Net B3 is made up of several CNN layers. Finally, there are the training phase layers, which are flattened, dropout, and predicted dense. Our dropout layer has a dropout rate of 0.3. The total number of parameters in the model was 11,386,829. Only 11,220,445 of them are trainable, with the remainder being nontrainable.

## 4. Result and Analysis

This section shows the performance of both the Mask-RCNN and Efficient Net B3 models. For model evaluation, we used different types of performance metrics for both the models.

### 4.1. Performance of Mask-RCNN Segmentation Model

For our segmentation model, we discovered the following final model MRCNN loss value in Table 1. We can see from the loss table that all the loss values are less than 0.5. As we all know, the better the model performs, the smaller the loss value is. We also determined our model's mAP and obtained an accuracy of about 93%, which is an excellent result.

**Figure 9:**
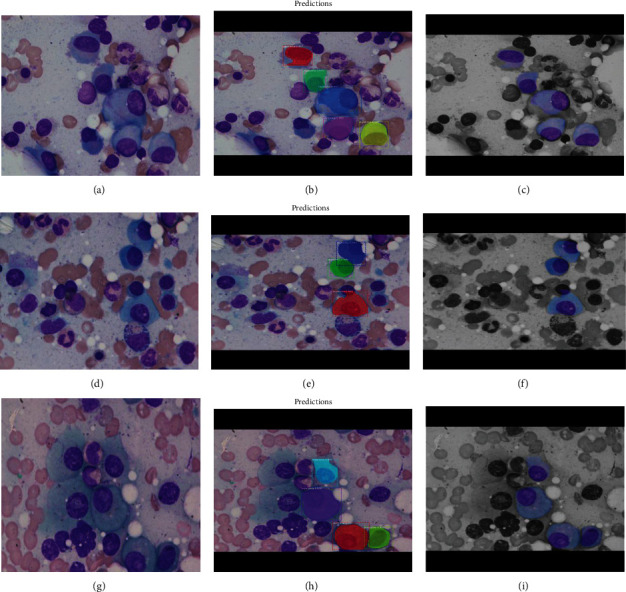
The above are the results of the segmentation steps: original picture (a), (d), (g); segmented image (B), (E), (H); and image after color splashing (C), (F), (I).

The MRCNN model was evaluated using validation data. The result is shown in Figure 9. Three experiments have been conducted here. Each run selects a random photo from the test data sets, makes a prediction, and returns three items: the bounding box, prediction class, and mask (cell regions).

Images in Figure 9(a), 9(d), and 9(g) are the real images, and 9(b), 9(e), and 9(h) are the myeloma detected images, where we can see the class name, bounding box, and finally, the mask of the detected area. The color splash of recognized pictures of 9(b), 9(e), and 9(h) is presented in the 9(c), 9(f), and 9(i) images for better understanding. The identified areas of myeloma cells in a picture are usually kept in RGB, whereas the remaining image is black and white. This way, the isolation of myeloma cells from the remaining cells can be indicated. In Figure 9, we can see that the myeloma cells are successfully masked and get isolated from them by keeping them in RGB format and making the rest of the portion of the image black and white.

### 4.2. Performance of Efficient Net CNN Model for Detecting Myeloma

**Figure 10:**
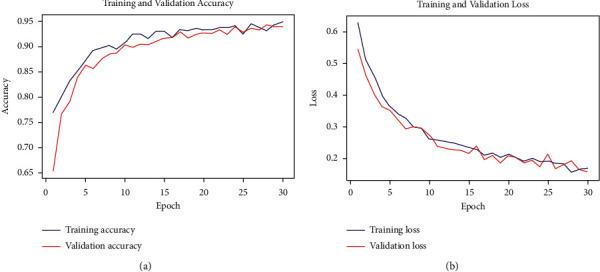
(a) Training and validation accuracy vs. epoch. (b) Training and validation loss vs. epoch.

After the model was built, the data set was split into training and validation with a ratio of 80 : 20. In all, there were 2080 training and 526 validation pictures. The learning rate (LR) was 0.0002 at the beginning, and the batch size was 32, resulting in a total of 2080/32 = 65 batched. Finally, the training began with 30 iterations, or epochs. Figures 10(a) and 10(b) show the model's training and validation accuracy and loss. At the first epoch of our model, the training accuracy started at just above 75% and the validation accuracy at just below 65%. After only five epochs, training and validation accuracy rose dramatically and became 87% and 85%, respectively. After epoch twenty, our model gets a little bit stabilized. The accuracy goes up and down for several epochs and finally ends with 94% for training and 93% for validation accuracy. We can see that the difference between training and validation is very small, which is good for our model.

Figure 10(b) is the graph of our training and validation loss. It is quite the opposite of accuracy. As the accuracy was raised, the loss decreased. Here, training and validation losses started at 65% and 58%, respectively. It rapidly decreased throughout the 30 epochs and finally reached below 20% at the end of the 30 epochs.

There are many accuracy metrics to see if our model is biased towards any specific prediction class. We must first determine the true negative, false positive, true positive, and false negative accuracy metrics. The precision rate is determined as demonstrated in equation (1):

(1) $$\begin{array}{c} \text{Precision}=\frac{\sum \text{Truew Positive}}{\sum \text{TruePostive}+\text{FalsePositive}}. \end{array}$$

The review rate is determined as demonstrated in equation (2):

(2) $$\begin{array}{c} \text{Recall}=\frac{\sum \text{Truew Positive}}{\sum \text{TruePostive}+\text{FalseNegative}}. \end{array}$$

The equation of the F1-Score is demonstrated in equation (3):

(3) $$\begin{array}{c} F1-\text{score}=\frac{2\times p\times R}{p+R}. \end{array}$$

**Figure 11:**
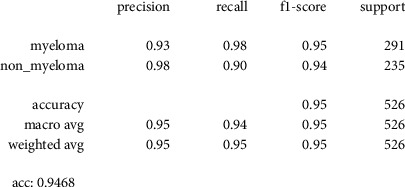
Accuracy matrices of our classification model.

Figure 11 shows the classification report of our trained model where recall, precision, *F*1-score, and accuracy are the same, which is 94.68%. Here, support is the number of samples we have in our validation data. For example, in the myeloma class, we have 291 samples and 235 samples in the nonmyeloma class. In total, we have 526 samples in our validation set.

**Figure 12:**
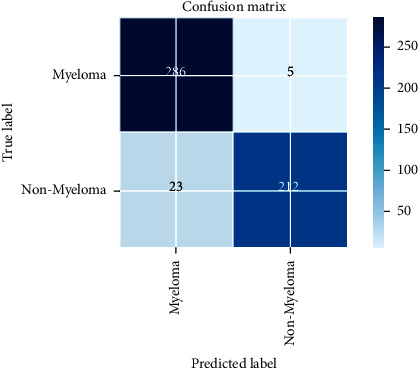
Confusion matrix of our trained model.

Figure 12 shows our model's confusion matrix where the values of true positive (TP) and true negative (TN) are 286 and 212, which are correct predictions. TP and TN are the values for which the accuracy of our model increases. On the other hand, false positive (FP) and false negative (FN) are the values for which the accuracy of a model decreases. We can see that with our trained model, the majority of the samples predicted their class correctly and had very few 5 FP and 23 FN values. TP and TN are the right detections. TP means that the actual label and predicted label are both predicted as positive, and TN means both predicted as negative. For example, if I give an image to test and the actual label of it is myeloma, and the prediction result is also myeloma, then it means a positive, and it will be counted in TP, and the reverse happens with TN. Where FP means the actual label was positive, but it predicts negative, and the opposite is true for FN.

**Figure 13:**
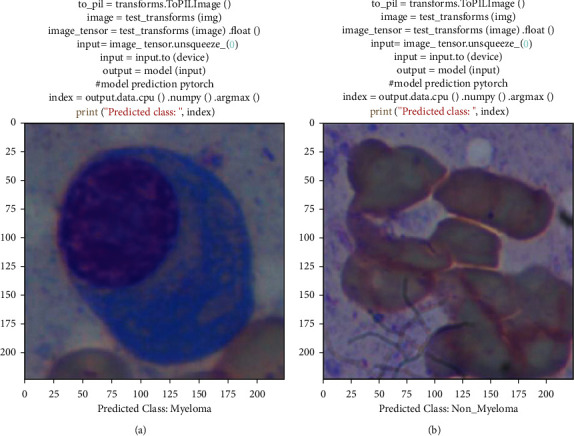
(a) Detection on single myeloma image. (b) Detection on single nonmyeloma image.

Figures 13(a) and 13(b) show the detection of myeloma and nonmyeloma in a single test image using our model and the code that was used.

In Figure 13(a), we used model (input) to feed a myeloma cell image into our model, and it returned an output of myeloma, indicating that the class was correctly predicted. Similarly, in Figure 13(b), we fed a nonmyeloma cell image to our model for prediction, and it correctly predicted nonmyeloma. So, for both of the class predictions, our model predicted successfully.

### 4.3. Model Comparison

In Table 2, our study's segmentation and classification models are compared with some previous work models with respect to mAP (mean average precision) for segmentation and accuracy for classification.

In Table 2, we can see that different types of methods have been used for the segmentation and classification of myeloma cancer cells. All of them give good overall accuracy. They are pretty close to each other. For the medical field, all of these accuracies are quite good. First of all, in a study [6], they did not find any segmentation accuracy metrics. They have shown only a classification accuracy of 93% using the KNN classifier. A study [8] has similar accuracy for classification, but they have also done segmentation using the watershed segmentation method and got an mAP of 90%. A study [15] got the lowest segmentation mAP of 89% and the highest classification accuracy of 94%. Finally, our research has higher accuracy than other studies for both segmentation and classification models. We got 93% mAP and 95% accuracy for segmentation and classification models, respectively. Although the increase is not that much, in medical science, a 1% increase is huge, so our model performs best with respect to it.

## 5. Conclusion

To determine the presence of leukemic cells, structural bone marrow and peripheral blood slide examinations are conducted. A pathologist will notice changes in the nuclei or cytoplasm of certain cells underneath a microscopic examination in order to recognize the aberrant cells in their unique forms and subtypes of leukemia. This classification can be used to predict the clinical course of the condition, and the patient should be treated accordingly. For an unexplained reason, the bone marrow generates many abnormal white blood cells in leukemia. Pathology is used to manually identify leukemia, which takes time and money due to pathology instruments' high cost. As a consequence, an automated method is employed for rapid and exact outcomes.

In this research, an automated method was used to create bone marrow smear images to recognize multiple myeloma cells and then detect those that were myeloma and nonmyeloma. The method started with data annotation and then the Mask-RCNN model was created. Finally, we trained the model with COCO weights. The final results of our Mask-RCNN model show an mAP of 93%. Then, we cropped all of the purple-colored myeloma and white-colored nonmyeloma cells for our second model and built a classification model to determine which are myeloma and which are not. The final result of our classification model is 94.68% for all the accuracy metrics such as precision, recall, *F*1-core, and accuracy. We have also found the confusion matrix for better visualization of our testing. Overall, our performance of both the segmentation and classification models is excellent.

For the classification model, we have already got 94.68% accuracy using transfer learning. In the future, if we increase our data set for myeloma and nonmyeloma, in our opinion, this accuracy may increase. For segmentation of the multiple myeloma cells, there is a lot of work that can be done. For example, several other segmentation methods are watershed, clustering, region-based, thresholding, and Otsus's segmentation. Where we can use all types of segmentation methods to recognize myeloma cells and compare them. Also, in the future, we can segment the nucleus of the multiple myeloma cells. We can also segment the nucleus and cytoplasm of myeloma cells and separate them from the actual myeloma cells. To extend our work, we will only need to annotate the images again with two classes: nucleus and cytoplasm.

## Figures and Tables

**Table 1:** Class, bounding box, and mask loss of our Mask-RCNN model.

Weights	Loss values
Bounding box loss	0.2985
Class loss	0.0908
Mask loss	0.3046

**Table 2:** Result comparison.

Reference	Method name	Segmentation mAP	Classification accuracy (%)
[4]	KNN classifier	None (%)	93
[6]	Watershed and SVM classifier	90	93
[13]	V-Net and KNN	89	94
Our study	Mask-RCNN and Efficient Net B3 CNN	93	95

## Data Availability

The data utilized to support these research findings are accessible online at https://wiki.cancerimagingarchive.net/pages/viewpage.action?pageId=52756988.
